# Bioinformatics Tools and Novel Challenges in Long Non-Coding RNAs (lncRNAs) Functional Analysis

**DOI:** 10.3390/ijms13010097

**Published:** 2011-12-23

**Authors:** Letizia Da Sacco, Antonella Baldassarre, Andrea Masotti

**Affiliations:** Gene Expression, Microarrays Laboratory, IRCCS-Bambino Gesù Children’s Hospital, P.za S.Onofrio 4, 00165 Rome, Italy; E-Mails: letizia.dasacco@opbg.net (L.D.S.); antonellabalda@libero.it (A.B.)

**Keywords:** non-coding RNAs, lncRNAs, genomics, bioinformatics, systems biology

## Abstract

The advent of next generation sequencing revealed that a fraction of transcribed RNAs (short and long RNAs) is non-coding. Long non-coding RNAs (lncRNAs) have a crucial role in regulating gene expression and in epigenetics (chromatin and histones remodeling). LncRNAs may have different roles: gene activators (signaling), repressors (decoy), *cis* and *trans* gene expression regulators (guides) and chromatin modificators (scaffolds) without the need to be mutually exclusive. LncRNAs are also implicated in a number of diseases. The huge amount of inhomogeneous data produced so far poses several bioinformatics challenges spanning from the simple annotation to the more complex functional annotation. In this review, we report and discuss several bioinformatics resources freely available and dealing with the study of lncRNAs. To our knowledge, this is the first review summarizing all the available bioinformatics resources on lncRNAs appeared in the literature after the completion of the human genome project. Therefore, the aim of this review is to provide a little guide for biologists and bioinformaticians looking for dedicated resources, public repositories and other tools for lncRNAs functional analysis.

## 1. Introduction

In the last few years, the advent of novel and high-throughput technologies to sequence the genome (next generation sequencing. NGS) has revealed that eukaryotes transcribe up to 90% of their genomic DNA [1]. However, only 1–2% of these transcripts encode for proteins [2,3], while the remaining large fraction of transcribed RNAs are classified as non-coding RNAs (ncRNAs) [4,5]. Furthermore, the fact that the majority of ncRNAs are expressed in a spatio-temporal manner at substantially lower levels than mRNAs, often exhibiting precise sub-cellular localization, suggests that these ncRNAs mainly fulfill regulatory functions and are more likely central components of an extensive RNA control network coexisting along with proteins [6].

**Figure 1:**
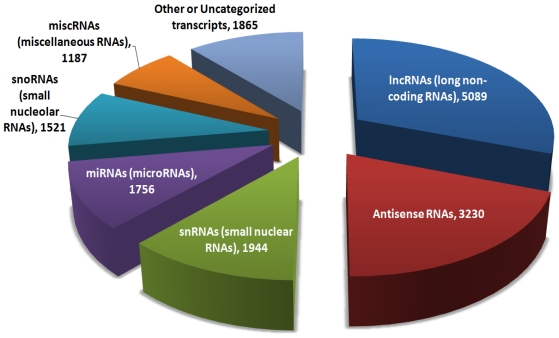
Long non-coding RNAs are just one of the many non-coding transcripts being annotated. The pie chart represents the major categories of the 16,592 non-coding RNAs. Adapted from [14].

Based on their size. ncRNAs can be classified as small (~18–31 nt), medium (~31–200 nt) and long (from 200 nt up to several hundred kb) transcripts [7,8]. While the small ncRNAs group contains small interfering RNAs (siRNAs), microRNAs (miRNAs) and Piwi interacting RNAs (piRNAs) and are localized in the cell cytoplasm, medium ncRNAs are mainly represented by small nuclear (snRNAs) and small nucleolar RNAs (snoRNAs) and reside in the cell nucleus (Figure 1).

Alternatively. ncRNAs can be functionally divided into housekeeping ncRNAs (*i.e.*, ribosomal, transfer, snRNAs and snoRNAs) playing crucial roles in many cellular processes, and regulatory ncRNAs (*i.e.*, miRNAs, piRNAs, siRNAs and lncRNAs) [9]. However, other regulatory ncRNAs such as promoter-associated RNAs (PARs) and enhancer RNAs (eRNAs) have been recently described and added to this increasing category [10–12].

Unlike small and medium ncRNAs, long non-coding RNAs (lncRNAs) are not highly conserved at the primary sequence level but they have been estimated to be ~17,000 in human and ~10,000 in the mouse genome [13].

Based on their genomic position, lncRNAs can be further catalogued into four categories: (i) sense or antisense (when the ncRNA overlaps one or more exons of another transcript on the same or opposite strand, respectively); (ii) bidirectional (when the expression of the ncRNA and that of a neighboring coding transcript on the opposite strand is initiated in close genomic proximity); (iii) intronic (when the ncRNA is derived from an intron of a second transcript); and (iv) intergenic (when the ncRNA is localized between two genes) [9].

All of these considerations emphasize the fact that the genome organization is more complex than previously thought. The conventional thinking of RNA as the messenger of the genetic information from DNA to protein translation is no longer acceptable. In fact, many lncRNAs can be primary transcripts for the production of shorter RNAs (such as miRNAs) [15], making a clear categorization of ncRNAs quite difficult. In eukaryotes, lncRNAs have been implicated in many biological processes with different functional roles such as X chromosome inactivation [16,17], genomic imprinting [18], sub-cellular structural organization [19,20], telomere [21] and centromere organization [22,23] and nuclear trafficking [24].

Finally, the different nomenclature found in many papers adds further complexity to understanding the nature and role of these ncRNAs and the retrieval of specific information. In fact, another subgroup of lncRNAs called “large intergenic” non-coding RNAs (or lincRNAs) has been described [25]. However, in a following paper the same group defines these ncRNAs as “large intervening” [26] while others call them simply “large” ncRNAs [27]. He *et al.* studied the “messenger-like non-coding RNAs” (ml-ncRNAs) as potential precursors of miRNAs [15]. Another definition has been reported by Zong *et al.* when they found “nuclear-retained” RNAs (nrRNAs) implicated in the modulation of gene expression by influencing chromatin modification, transcription and posttranscriptional gene processing [28]. According to the authors’ feeling, we agree that our understanding of the functional role played by ncRNAs is like “a tip of an iceberg” and that the unraveling of ncRNAs’ functions and of their interplay with other biological actors, deserves further experimental investigations and focused bioinformatics efforts.

Although next generation technologies in RNA sequencing (RNA-Seq) allowed the identification of thousands of lncRNAs with an unprecedented throughput, only a few of them have been completely characterized from a functional point of view. In fact, determining the function of individual lncRNAs still remains challenging [11]. Computational tools allowing researchers to know what they are looking for, if they are finding something new, the name of what they are finding or to better characterize the potential functions of these lncRNAs, would therefore facilitate the unraveling of the biological role and to emphasize the significance of this group of ncRNAs in a variety of systems and diseases.

The aim of this review is focused on those computational approaches and bioinformatics resources available to researchers dealing with lncRNAs functional analysis. We also present a brief overview of the mechanisms of action of lncRNAs as transcriptional and epigenetic regulators.

## 2. The Four Main Roles of Long Non-Coding RNAs

The main features and properties of long non-coding RNAs will be briefly presented in the following paragraph. We decided to mention briefly the four major roles of lncRNAs in order to treat in more detail the bioinformatics tools and resources available to researchers involved in the study, functional analysis or simply annotation of known (and unknown) lncRNAs.

**Figure 2:**
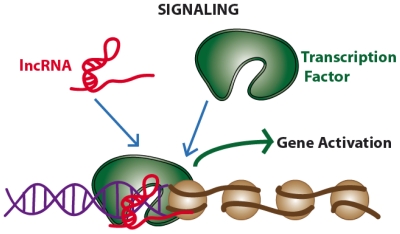
Long non-coding RNAs acting as gene activators (signaling archetype).

**Figure 3:**
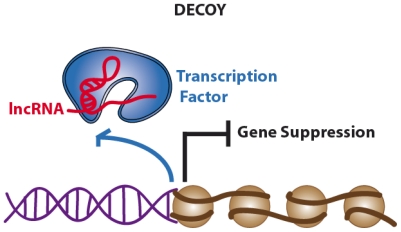
Long non-coding RNAs acting as gene suppressors (decoy archetype).

**Figure 4:**
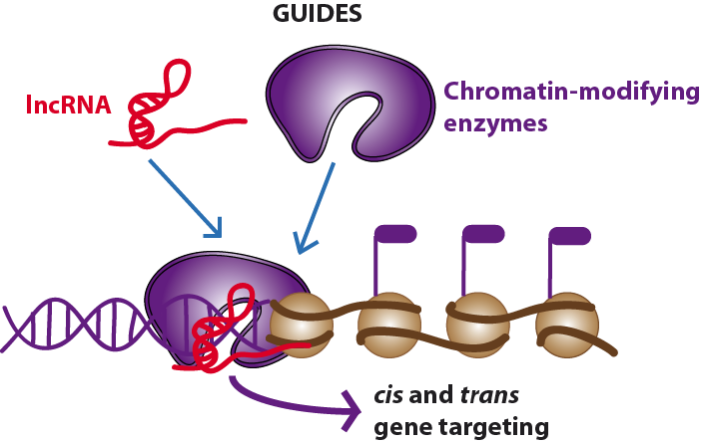
Long non-coding RNAs acting as *cis* and *trans* gene expression regulators (guide archetype).

**Figure 5:**
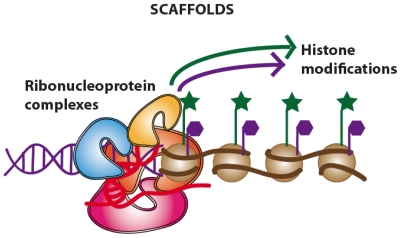
Long non-coding RNAs acting as chromatin modificators (scaffold archetype).

LncRNAs show a low level of sequence conservation, they generally have a 7-methylguanosine cap at 5′ end, they can terminate with or without a poly(A) chain at their 3′ end [26,29,30] and they have well-defined sites for binding of transcription factors (*i.e.*, NF-kB) in their promoter regions [31]. Most lncRNAs are expressed in a tissue-specific pattern and transcribed by RNA polymerase II (RNA Pol II), as also suggested by the presence of trimethylation of lysine residue K4 of histone H3 (H3K4me3) in the promoter region and H3K36me3 along the transcript, the so called “chromatin signature (a K4-K36 domain)” [26]. However, several lncRNAs are also transcribed by RNA Pol III [32,33]. As lncRNAs reside in the nucleus, their functions span from the regulation of transcription and modeling of nuclear architecture, to telomere biology and chromosomal dynamics [34]. According to the authoritative review by Wang and Chang published this year, lncRNAs can be categorized in four main categories or archetypes: signals, decoys, guides and scaffolds (Figures 2–5) [35].

While the mechanism of signals and decoys deals with gene activation or suppression (Figures 2 and 3) respectively, guides can recruit chromatin-modifying enzymes to regulate the expression of genes, either in *cis* (near the site of lncRNA production) or in *trans* (distant genes) through a phenomenon called “transvection” (Figure 4). Finally, scaffolds can bring together multiple proteins to form ribonucleoprotein (RNP) complexes (Figure 5) and these lncRNA-RNP systems stabilize nuclear structures or signaling complexes acting on chromatin and determining histone modifications.

To a first approximation, we can say that the first two types of functions (signaling and decoys) deal with gene expression and regulation, while the latter two (guides and scaffolds) deal with epigenetic modifications. However, authors emphasized that each lncRNAs may have several functions belonging to different functional types without the need to be mutually exclusive; rather, lncRNAs may develop complex functions exploiting a combination of different molecular mechanisms.

### 2.1. Signaling. Long Non-Coding RNAs Acting as Gene Expression Enhancers or Repressors

Ørom *et al.* recently characterized over a thousand lncRNAs expressed in multiple cell lines (*i.e.*, fibroblasts, keratinocytes and HeLa cells). They found that depletion of certain number of lncRNAs led to a consequent decreased expression of their neighboring protein-coding genes such as the master regulator of hematopoiesis, SCL (also called TAL1), Snai1 and Snai2 [12]. With their study they outlined that even a small set of lncRNAs can act as gene expression enhancers in various human cell lines.

There are other examples in the literature addressing that the transcription of lncRNA can lead to an active silencing at their respective genomic locations, especially in imprinting mechanisms. Imprinting is an epigenetic regulatory mechanism that in mammals leads to silencing of autosomal genes in one of the two alleles, one inherited from the mother and one from the father. LncRNAs such as Kcnq1ot1 and Air, which map to the Kcnq1 and Igf2r imprinted gene clusters, respectively, mediate the transcriptional silencing of multiple genes by interacting with chromatin and recruiting the chromatin-modifying machinery [18,36]. Also Xist is another well-known lncRNA that plays an essential role in chromosome X inactivation (XCI) in female cells [37,38]. In fact, during development, the expressed lncRNA Xist “covers” the X chromosome from which it is transcribed, leading to a generalized repression of gene expression.

However, lncRNAs may be also involved in stress events such as in the response to DNA damage as recently reported by Huarte *et al.* [39]. LincRNA-p21 is a long intergenic non-coding RNA located upstream of CDKN1A gene, that has been reported to have an important regulatory role as transcriptional repressor in the canonical p53 pathway and to trigger apoptosis. In this regulatory mechanism, p53 directly induces lincRNA-p21 expression most likely through the direct interaction of p53 itself to the lincRNA-p21 promoter region, while reduction of lincRNA-p21 has been reported to increase the expression of numerous p53-repressed transcripts [39]. Besides, the mammalian CDKN1A promoter has been reported to be involved in several lncRNAs transcription upon DNA damage [40]. PANDA, one of these lncRNAs, is expressed only in p53-positive cells (p53 bind to the CDKN1A locus) and interacts with the transcription factor NF-YA down-regulating the expression of pro-apoptotic genes and enabling cell-cycle arrest. PANDA may also act as a decoy, as better described in the following paragraph.

### 2.2. Decoys. Long Non-Coding RNAs Acting as Molecular Sinks

The lncRNA PANDA has either signal and decoy functions, as already mentioned above. Having a decoy function means that the lncRNA, once transcribed, binds to a target protein and keeps it apart, preventing the exploitation of its function. In this way, lncRNAs act to repress the action of RBPs, transcription factors, chromatin modifiers, or other regulatory factors. LncRNA PANDA is very sensitive to DNA damage and inhibits the expression of apoptotic genes through direct binding to (and sequestering of) NF-YA transcription factor [40]. When the DNA damage is low or moderate, the cell survives. Interestingly, a subset of human breast cancers overexpresses PANDA, while PANDA depletion can sensitize cells to chemotherapeutic agent, suggesting novel potential clinical applications.

The telomeric repeat-containing RNA (TERRA) is another lncRNA involved in telomeric heterochromatin assembly. It is transcribed from telomeres, the DNA-protein complexes located at the physical ends of eukaryotic chromosomes, essential for chromosome stability [41]. TERRA has been demonstrated to have mainly two functions: to interact with telomerase RNA through a repeating sequence [42], and to bind to the telomerase reverse transcriptase (TERT) protein subunit. TERRA has been shown to bind and sequester telomerase retaining telomerase near the telomeric 3′ end but at the same time, inhibiting its action [42]. TERRA is however transcribed in a cell-cycle dependent manner (with the highest levels in G1 phase and the lowest in S phase), suggesting that telomerase may be consequently regulated.

### 2.3. Guides and Scaffolds. Long Non-Coding RNAs Acting as “Molecular Assemblers”

When lncRNAs bind to proteins and direct the localization of RBP complex to specific target for chromatin modification, we can assume that they are working as “molecular guides”. As previously mentioned, lncRNAs can guide changes in gene expression either in *cis* or in *trans* in a way difficult to predict if one takes into account only their sequences.

This mode of action is quite complicated also by the fact that lncRNAs may contact several effector molecules such as the trithorax group proteins (TxG), the polycomb group proteins (PcG), and the common set of transcription factors [35]. This guiding mode of action is intimately linked to the scaffold function, that is, when other factors are recruited to form a “molecular platform” for chromatin modification leading to the modulation of central signaling events [43]. To exploit this function, lncRNA should have different domains for distinctly binding different effector molecules at the same time and in timely and spatially manner. Therefore, it appears clear that the understanding of the ways by which lncRNAs are able to form these complex scaffolds is crucial and not fully achieved so far.

To cite only a couple of examples of this mode of action, we may recall two processes: the telomerase assembly and the gene repression through the polycomb complex PRC2. In the former process, the reverse transcriptase telomerase contributes to maintain genome stability by adding telomeric DNA repeats to chromosome ends. To exploit this catalytic activity, telomerase requires that two subunits come into close contact: the telomerase RNA component TERC and the catalytic transcriptase TERT. Additionally, other components or accessory proteins can take part to the process [44].

The protein complex PRC2 (Polycomb Repressive Complex 2) has histone methyltransferase activity and transfers three methyl groups to histone H3 on lysine 27 (H3K27me3). The histone methylation leads to a transcriptionally silent chromatin. It has been shown that the lncRNA HOTAIR binds to this complex through an upstream sequence of 300 nt from the beginning of the 5′ edge [45] repressing gene expression [46]. At the same time, the downstream sequence located 700 nt from the 3′ end of HOTAIR has been found to interact with a second protein complex recruited to demethylate histone H3 on K4 and containing LSD1, CoREST, and REST [45]. These recent findings emphasize the importance of HOTAIR (and of other lncRNAs) in the chromatin modification processes leading to suppression of gene expression and indicate that these complex RNA-protein interactions should be studied in details in order to fully understand the potential mechanisms in which they are involved.

Therefore, it appears clear that bioinformatics and systems biology approaches have a crucial role in assisting researchers to shed a light on those biological processes where lncRNAs are involved and where most of the mechanisms have still to be understood. For this reason, we decided to review the tools (software, database and other utilities) available to researchers dealing with the interpretation of the molecular mechanisms underlying this complex emerging field.

## 3. Functional Analysis of Long Non-Coding RNAs (lncRNAs)

Several bioinformatics resources are available to researchers for different purposes and they include database and repositories, annotation tools and other software. Table 1 summarizes the resources available so far.

### 3.1. Databases and Public Repositories

One of the first database developed to give information on ncRNAs with documented or possible regulatory functions has been developed by Barciszewski’s group in 2003 [47]. Their Noncoding RNA database (http://biobases.ibch.poznan.pl/ncRNA/) was the first repository containing nucleotide sequences (retrievable in FASTA format), short descriptions of the activities of particular ncRNAs, GenBank accession numbers and literature references. However, at that time, the total number of unique mammalian ncRNAs in the database was <40 excluding homologs and miRNAs. Currently, the database includes over 30,000 individual sequences from 99 species of Bacteria, Archaea and Eukaryota. The primary source of sequences included in the database was the GenBank and additional annotation information for mouse and human ncRNAs were derived from FANTOM3 database and H-inviational Integrated Database of Annotated Human Genes version 3.4, respectively.

Another database developed the same year was Rfam (http://www.sanger.ac.uk/Software/Rfam/) a collection of multiple sequence alignments and covariance models representing non-coding RNA families [48]. The first release of Rfam (1.0) contained over 50.000 ncRNA genes belonging to 25 families. After integration with more specialized RNA databases such as miRBase, IRESite, Pseudobase, snoRNABase, the plant snoRNA database, TransTerm and the Yeast snoRNA database, authors envisage that the next version (Rfam 9.1) will contain more than 700 entirely new families, reaching a total of more than 1300 [49].

In year 2005, Mattick’s group reported an implementation of a previous ncRNAs catalogue, developing RNAdb (http://research.imb.uq.edu.au/RNAdb), a comprehensive mammalian noncoding RNA database containing over 800 unique experimentally studied ncRNAs, associated with diseases and/or developmental processes [50]. This database was further implemented in 2007 with the RNAdb 2.0 [51] where the authors provided also nucleotide sequences and annotations for tens of thousands of non-housekeeping ncRNAs, including a wide range of mammalian microRNAs, small nucleolar RNAs as well as ncRNAs predicted on the basis of structural features and alignments.

Another curated dataset, the H-Invitational Database (H-InvDB) (http://www.h-invitational.jp/), resulted from the joint efforts of many researchers involved in the Human Full-length cDNA Annotation Invitational project [52]. H-InvDB (release 3.4, August 2006), produced by the “Genome Information Integration Project” (2005–2008), contains more than 1700 putative ncRNAs defined by the absence of any open reading frame and by not belonging to the pseudogene classification.

Over the years, several other databases emerged to fill the gaps of categorizing other ncRNAs such as SRP RNAs, tmRNAs or RNase P RNAs and other ncRNAs named according to cellular localizations (*i.e.*, snRNAs, snoRNAs or scRNAs), to functions (*i.e.*, package, guide or transfer-messenger RNAs) or to their sedimentation coefficients (*i.e.*, 6S RNA, 5.3S RNA, *etc.*). Therefore, to establish a common and uniform classification system, the ncRNA database NONCODE (http://www.noncode.org, http://noncode.bioinfo.org.cn) has been created [54]. The first release of NONCODE (v1.0) contained 5339 non-redundant sequences from 861 organisms, including eukaryotes, eubacteria, archaebacteria, virus and viroids. In the following years, a significant growth in the amount of data on ncRNAs led to the NONCODE v.2.0, where the number of collected ncRNAs reached over 206226 non-redundant sequences from 861 organisms [55]. In this version, other novel classes of ncRNAs, such as Piwi-interacting RNAs (piRNAs), stem-bulge RNAs (sbRNAs) and snRNA-like RNAs (snlRNAs) [64] have been included together with other unclassified ncRNAs. To date, NONCODE has reached the version 3.0 and now contains 42,3976 public sequences from 1239 organisms covering all kingdoms of life, including vira and viroids. One of the appealing features with this database, is the possibility to obtain functional information on a lncRNA of interest. In fact, NONCODE integrates a classification system, the “process function class” or PfClass, that is based on the cellular process and function in which a given ncRNA is involved. Therefore, PfClass is a unified classification system giving an output of concise functional annotations for a certain ncRNA.

By integrating different available databases such as FANTOM3 [65], H-invDB rel. 5.0 [53], miRBase v10.0 [66], NONCODE v1.0 [54], Rfam v8.1 [48], RNAdb v2.0 [51], snoRNA-LBME-db rel. 3 [67], and Gene Expression Omnibus (GEO) [68], another Japanese group generated a platform for mining/annotating functional RNA candidates from non-coding RNA sequences, that they called fRNAdb (http://www.ncrna.org/frnadb) [57]. fRNAdb is a database providing a support for computational analyses related to RNA secondary structure motif discovery, EST support evaluation, *cis*-regulatory element search and protein homology search. Moreover, the fRNAdb interface is linked to a customized UCSC Genome Browser (RNA-specific custom tracks). The updated version fRNAdb 3.0 supports two important tasks: annotation of anonymous RNA transcripts and discovery of novel non-coding RNAs [58]. Interestingly, the last version of fRNAdb not only expanded the number of sequences from 13,693 to 509,795, but, as in the case of the NONCODE database, it also integrated sequence ontology classification (SO, http://song.sourceforge.net/), keyword search function and Blast search service.

As previously described, in mammals there are a series of non-coding RNAs mono-allelically expressed in a parent-dependent manner, that have been called “imprinted” ncRNAs [69]. Increasing evidences suggest that dysregulation of imprinted ncRNAs are implicated in many human diseases such as Prader-Willi syndrome (PWS), Beckwith-Wiedemann syndrome (BWS), Silver-Russell syndrome (SRS), transient neonatal diabetes mellitus (TNDM), and various tumors [70–72]. At present, this category of imprinted ncRNAs includes small nucleolar RNAs (snoRNAs), microRNAs (miRNAs), small interfering RNAs (siRNAs), Piwi-interacting RNAs (piRNAs), antisense ncRNAs, and mRNA-like ncRNAs [73]. To reduce the scattering of data found on the literature or in different available databases, Zhang et al. systematically collected all the information of mammalian imprinted ncRNAs in a comprehensive database called ncRNAimprint [59]. This database contains 7094 entries where the majority are represented by piRNA (6612), followed by microRNAs (187), snoRNAs (129), siRNAs (107) and antisense ncRNAs (26). For mammalian species, only 33 records for mRNA-like ncRNAs are currently available (http://rnaqueen.sysu.edu.cn/ncRNAimprint).

Another public repository is represented by the Noncoding RNA Expression Database (NRED) (http://jsm-research.imb.uq.edu.au/NRED), which provides gene expression information for thousands of long ncRNAs in human and mouse [60]. NRED is a multifaceted repository since it integrates also evolutionary conservation, secondary structure evidence, genomic context links and antisense relationships for represented lncRNAs. Therefore, NRED gives also useful information for characterizing lncRNAs based on these criteria. An updated version of this repository (NRED2) has been expected for 2011.

The NRED resource has been linked to another reference database for lncRNAs called lncRNAdb [13]. This database collects all the lncRNAs associated with any of the biological functions in eukaryotes, or those mRNAs with a regulatory role (http://www.lncrnadb.org/). LncRNAdb includes information about sequences, structural information, genomic context, expression, subcellular localization, conservation, functional evidence and other relevant information and is linked to the UCSC Genome Browser for visualization and Noncoding RNA Expression Database (NRED) for expression information from a variety of sources. The database contains over 150 lncRNAs identified in 60 different species and most of them (~75%) are from mammals, the more intensively studied class.

In order to simplify the challenging determination of lncRNAs function, Moran et al. described the development of a novel computational approach that consisted in the integration of RNA-seq data with available annotation resources [61]. Authors characterized lncRNAs using multiple features, including sequence, structural, transcriptional, and orthology information. They obtained a stringent set of 4662 human lincRNAs loci (14,353 transcripts), 2798 of which (~60%) were not identified by RefSeq, UCSC, and GENCODE. Then, the authors generated a reference catalog of 8195 human lincRNAs based on integrating RNA-seq data from 24 tissues and cell types with publicly available transcript annotations. They also found that the expression of lncRNAs is tissue-specific and linked to the expression of their neighboring genes. These results have been collected into the Human Body Map lincRNAs (http://www.broadinstitute.org/genome_bio/human_lincrnas/). This integrated and comprehensive reference catalog could help to analyze the global properties of lncRNAs and facilitate further studies on functional classification of these non-coding genes.

### 3.2. Annotation Tools and Other Bioinformatics Tools

In the last few years we assisted at an exponential growth of genome-wide expression studies as a result of an extensive use of microarray technology. However, none of these studies addressed the problem to take into proper consideration the location of the measuring probes in the context of the currently known genomes and transcriptomes. Besides, the number of noncoding genes and their associated isoforms increases continuously. Therefore, to meet the need to develop new tools combining genomic and transcriptomic information and to provide a clear mapping of expression probes to current genomic annotations, the Genomic and Transcriptomic Explorer (GATExplorer) web platform (http://bioinfow.dep.usal.es/xgate/) has been developed [62]. GATExplorer is a web platform and a database integrating a gene loci browser with nucleotide level mappings of oligo probes from expression microarrays. It allows an interactive exploration of gene loci, transcripts and exons of human, mouse and rat genomes, and shows the specific location of all “mappable” Affymetrix microarray probes and their respective expression levels in a broad set of biological samples. The web site allows visualization of probes in their genomic context together with any associated protein-coding or noncoding transcripts. GATExplorer integrates data from Ensembl, RNAdb, Affymetrix and GeneAtlas. This tool is very useful when researchers are dealing with gene discovery, because considering expression at the nucleotide level rather than at the gene level, it allows to detect expression signals from novel entities. GATExplorer provides the means to undertake a higher resolution analysis of microarray data and potentially to extract considerably more detailed and biologically accurate information from existing and future microarray experiments.

Another web interface, the non-coding RNA Function ANnotation server (ncFANS) (http://www.ebiomed.org/ncFANs/), recently emerged in the panorama of computational tools dedicated to the analysis of lncRNAs [63]. The tool ncFANs has been developed for functional annotation of specific lncRNAs, both in mouse and in human, through the re-annotation of Affymetrix array data, finally giving the possibility to re-analyze microarray data already acquired for other experiments. To annotate the lncRNAs function, ncFANs provides two alternative strategies: the first employs a coding-noncoding gene co-expression (CNC) network [74], the other identifies conditionrelated differentially expressed lncRNAs.

### 3.3. Biological and Bioinformatics Approaches for lncRNA Discovery

Genomic projects over the past decade have stably confirmed the presence of many thousands of non-coding transcripts in mammals [75]. However, the understanding of lncRNAs function is still partially unknown. Two main challenges prevent this knowledge: (1) to identify likely functional lncRNAs and (2) to infer lncRNAs putative function in hypothesis-driven experiments. Guttman *et al.* recently developed an efficient method to create genome-wide chromatin-state maps, using chromatin immunoprecipitation followed by massively parallel sequencing (ChIP-Seq) [76]. Genes actively transcribed by RNA Pol II are marked by trimethylation of lysine 4 of histone H3 (H3K4me3) at their promoter and trimethylation of lysine 36 of histone H3 (H3K36me3) along the length of the transcribed region (feature called “K4–K36 domain”). The identification of K4–K36 structures residing outside known protein-coding gene loci, allowed the systematic discovery of functional lncRNAs [26]. The authors created an RNA expression compendium of both lncRNAs and protein-coding genes across a wide range of tissues to infer lncRNAs putative function that they assessed experimentally. This analysis revealed numerous sets of lncRNAs associated with distinct and diverse biological processes. Moreover, Guttman *et al.* found that chromatin structure can lead to the identification of sets of lncRNAs showing a high degree of evolutionary conservation, therefore biologically functional. With their approach, the functional roles for 150 lncRNAs have been obtained, and they have also predicted the pathways for almost 85 lncRNAs. Therefore, the authors demonstrated that the pipeline they reported can be efficiently employed for inferring putative roles for lncRNAs.

To solve the computational challenges of identification of diffuse signals from the chromatin immunoprecipitation and high-throughput massively parallel sequencing (ChIP-Seq) technology, and to suggest a new method respect to those currently available, Garmire et al. presented a novel global clustering approach to enrich diffuse CHIP-Seq signals of RNA polymerase II and histone 3 lysine 4 trimethylation (H3K4Me3) and applied it to identify putative lncRNAs in macrophage cells [77]. The authors implemented an iterative global-clustering-over-linear-separator (GCLS) algorithm to reconstruct the most correct transcription units and they found a total of 374 putative lncRNAs in macrophages under the “no treatment” condition and 189 lncRNAs under LPS treatment.

**Figure 6:**
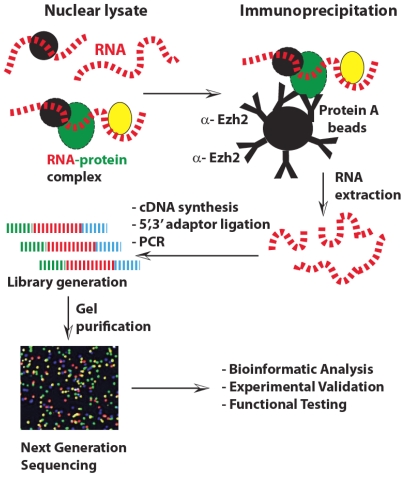
Rip-seq technology. After cell lysis an immunoprecipitation step is required to isolate RNA bound to Polycomb proteins. Retrotranscription to cDNA and ligation with suitable adapters are required prior to next generation sequencing. Bioinformatics analysis and further validation led to the identification of novel lncRNAs.

Finally, Zhao et al. elaborated a novel method to characterize lncRNAs starting from the concept that RNA-mediated recruitment is especially attractive for conserved Polycomb proteins controlling many aspects of development [78]. Mammalian PRC2 contains four core subunits, Eed, Suz12, RbAp48, and the catalytic Ezh2. Interestingly, several PRC2 subunits have potential RNA-binding motifs [79] while a recent work identified several short RNAs of 50–200 nt as candidate PRC2 regulators [80]. Therefore, they developed the RIP-seq technology to capture a genome-wide pool of long transcripts (>200 nt) associated with PRC2. This method consists in capturing the genome-wide pool bound to PRC2 by combining native RIP [81] and RNA-seq [82] in a combined protocol that they called “RIP-seq” (Figure 6).

Authors demonstrated that RIP-seq technology can be employed to identify RNA cofactors for other chromatin modifiers, and that different cell types might have different transcriptomes, consistent with their developmental profiles. Because chromatin modifiers such as PRC2 play a central role in several biological conditions, a genome-wide profile of regulatory RNAs represents a valuable tool in treating and diagnosing several diseases.

## 5. Conclusions

The discovery of long non-coding RNAs posed several challenges to researchers dealing with these “junk” molecules. The number of these small and long transcripts continued to increase since the last several years. Various databases has emerged to meet the need of rationalizing the information and to help researchers in their annotation and description work. There are other challenges still open, such as finding a common nomenclature and constructing a general functional ontology. Surely, in the future we will assist to an explosion of information about these non-coding RNAs. since to date very little is known about their precise role in many biological mechanisms and disease pathogenesis. We think that the advent of the next generation sequencing coupled to bioinformatics approaches will lead to a detailed description of the role of long non-coding RNAs and to the discovery of an unexplored world.

## Figures and Tables

**Table 1:** Bioinformatics resources (database, public repositories, annotation tools and other software) are summarized along with web links and year of publication. Resources are listed as described in the review.

Bioinformatics Resources	Year	Web Link	Reference
***Databases and public repositories***			
NcRNAdb	2003	http://biobases.ibch.poznan.pl/ncRNA	[47]
Rfam	2003	http://www.sanger.ac.uk/Software/Rfam	[48,49]
RNAdb	2005	http://research.imb.uq.edu.au/RNAdb	[50]
RNAdb 2.0	2007	http://research.imb.uq.edu.au/RNAdb	[51]
H-InvDB	2004	http://www.h-invitational.jp	[52]
H-InvDB rel 5.0	2005	http://www.h-invitational.jp	[53]
NONCODE	2005	http://www.noncode.org	[54]
NONCODE v2.0	2008	http://www.noncode.org	[55]
NONCODE v3.0	2011	http://www.noncode.org	[56]
fRNAdb	2007	http://www.ncrna.org/frnadb	[57]
fRNAdb 3.0	2009	http://www.ncrna.org/frnadb	[58]
ncRNAimprint	2010	http://rnaqueen.sysu.edu.cn/ncRNAimprint/	[59]
NRED	2009	http://jsm-research.imb.uq.edu.au/NRED	[60]
lncRNAdb	2011	http://www.lncrnadb.org/	[13]
Human Body Map lincRNAs	2011	http://www.broadinstitute.org/genome_bio/human_lincrnas/	[61]
***Annotation tools and other bioinformatics tools***			
GATExplorer	2010	http://bioinfow.dep.usal.es/xgate/	[62]
ncFANs	2011	http://www.ebiomed.org/ncFANs/	[63]
